# Paradigms and Success Stories of Natural Products in Drug Discovery Against Neurodegenerative Disorders (NDDs)

**DOI:** 10.2174/1570159X21666230105110834

**Published:** 2023-11-06

**Authors:** Sukhwinder Singh, Shivani Chib, Md. Jawaid Akhtar, Bhupinder Kumar, Pooja A. Chawla, Rohit Bhatia

**Affiliations:** 1 Department of Pharmaceutical Chemistry and Analysis, ISF College of Pharmacy Moga, Punjab, 142001, India;; 2 Department of Pharmacology, ISF College of Pharmacy Moga, Punjab, 142001, India;; 3 Department of Pharmaceutical Chemistry, College of Pharmacy, National University of Science and Technology, PO620, PC 130 Azaiba, Bousher, Muscat, Oman;; 4 Department of Pharmaceutical Sciences, HNB Garhwal University, Chauras Campus, Srinagar, Garhwal, Uttarakhand, 246174, India

**Keywords:** Neurodegenerative disorders, natural products, Alzheimer’s disease, Parkinson’s disease, drug discovery, clinical trials

## Abstract

Neurodegenerative disorders (NDDs) are multifaceted complex disorders that have put a great health and economic burden around the globe nowadays. The multi-factorial nature of NDDs has presented a great challenge in drug discovery and continuous efforts are in progress in search of suitable therapeutic candidates. Nature has a great wealth of active principles in its lap that has cured the human population since ancient times. Natural products have revealed several benefits over conventional synthetic medications and scientists have shifted their vision towards exploring the therapeutic potentials of natural products in the past few years. The structural mimicking of natural compounds to endogenous ligands has presented them as a potential therapeutic candidate to prevent the development of NDDs. In the presented review, authors have summarized demographical facts about various NDDs including Alzheimer’s disease (AD), Parkinson’s disease (PD), Huntington’s disease (HD) and various types of sclerosis in the brain. The significant findings of new active principles of natural origin along with their therapeutic potentials on NDDs have been included. Also, a description of clinical trials and patents on natural products has been enlisted in this compilation. Although natural products have shown promising success in drug discovery against NDDs, still their use is associated with several ethical issues which need to be solved in the upcoming time.

## 1 INTRODUCTION

Healthcare professionals attribute human health and well-being to cardinal activities of translational science including drug discovery and development. Machine learning [1], artificial intelligence [2], structure-based drug discovery [3], drug repurposing [4], and *in-silico* methods [5] are being deployed to get a move on drug discovery and development. The development in pharmacotherapy over years has been largely supported by natural products and their structural modifications. The broad structural diversity and multidimensional chemical structures of isolated natural products make them a distinctive fount-head for lead molecules [6].

Degenerative nerve diseases, also known as neurodegenerative disorders (NDDs) may be caused genetically or due to chemical or toxic agents, viruses, or medical conditions including stroke, tumor, and alcoholism. NDDs include, but are not limited to Alzheimer’s disease (AD), Huntington’s disease (HD), Parkinson’s disease (PD), Friedreich ataxia (FA), and Amyotrophic lateral sclerosis (ALS); AD and PD are the most common NDDs [7]. Oxidative stress has been identified and pointed out the most as a cause of brain impairment in neurodegenerative diseases [8-10]. In consonance with the recent report by Alzheimer’s disease association, as many as 6.2 million Americans are currently living with Alzheimer's disease among which 72% are 75 and above in age. Moreover, deaths have increased by 145% between 2000 and 2019 [11, 12]. Surprisingly, an estimated 1.2 million people could be living with PD in the United States by 2030 [13]. The prevalence of HD has been raised in the US (13.7/10000 persons) [14], UK [15], Italy (3.9/100000) [16], and countries across the globe. Besides, the prevalence of Friedreich ataxia in Central Europe has been reported to be 1:20000 to 1:50000 [17], and the incidence of ALS to be between 0.6 and 3.8 per 100000 person-years, which was higher in Europe ranging from 2.1 to 3.8 per 100 000 person-years [18].

Despite a huge number of synthesized drugs for numerous diseases, the customary problems encountered with synthetic drugs include resistance and toxicity [19]. Moreover, the total expense size of the drug journey from synthesis to marketing has shifted largely the interests of researchers to drug repositioning and repurposing [20]. Even with significant advancements in the pharmaceutical field, currently employed drugs in contemporary therapy provides symptomatic treatment and focus on providing improvement in motor ad non-motor symptoms [21-24]. However, increasing the number of computational web resources, strengthening the toxicity predicting algorithms, upgrading virtual screening methods, and better lead identification and optimization are still major challenges in drug discovery [25].

Contrastingly, natural products have taken a grip on the scientific interests in the discovery of lead molecules due to the vast range of scaffolds with multifaceted physicochemical and therapeutic activities and budget research. The historical use of natural products in form of concentrated concoctions, powders, and dried parts for the treatment of ailments has provided intellectual stimulus or molecular templates for the synthesis of about fifty percent of conventionally synthesized drugs [26]. Surprisingly, studies have revealed people's bias for natural drugs over synthetic ones [27, 28]. Notwithstanding, the multidimensional nature of drug discovery requires the establishment of safety, efficacy, and pharmacokinetic data of natural drug products which bring into use the advent of modern technologies such as artificial intelligence and microfluidics. These technological and computational promotions have opened new avenues to process multiplexed natural products and utilize their scaffolds to innovate and derive useful drug molecules [29].

In the current review, the authors have reviewed and prepared a compile-up of NDDs, the importance of natural products in their treatment, and success stories including patents, clinical trials, and marketed formulations of the natural products for NDDs in the most effective way.

## 2 NEURODEGENERATION AND DISORDERS

Endoplasmic reticulum stress and intensifying neuronal loss are the common characteristics of neurodegeneration. NDDs, by disrupting the communication between brain-spinal cord neural cells and muscles affect many bodily activities including balance, breathing, cardiac functions, talking, walking, and other movements [30]. Although, NDDs have been classified based on cognitive disorders, clinical examinations, extrapyramidal and pyramidal movement disorders, Neuropathic evaluation at autopsy is considered to be the current diagnostic gold standard that classifies NDDs into four main types *viz*. amyloidosis, tauopathies, synucleinopathies, and TDP-43 Proteinopathies [31]. At least some characteristics of amyloid protein are found in almost all NDDs, amyloid-like filamentous aggregates are found within neuronal and glial cytoplasm. Moreover, extracellular deposits of amyloid can also be found as plaques in brain parenchyma or in the form of amyloid angiopathy in the walls of blood vessels. The presence of β-Amyloid (Aβ), a proteolytic product of amyloid precursor protein deposits in parenchyma as senile plaques and neuronal tau inclusions is an elucidating feature of AD [32, 33].

NDDs having a pathological accumulation of tau proteins (microtubule-associated phosphoprotein) are classified as tauopathies [34] and generally subdivided into primary and secondary tauopathies. In the bargain, synucleinopathies are characterized by the aggregation of α-synuclein (presynaptic protein), a protein constituent of Lewy bodies that are found in PD. Also, TDP-43, a nuclear protein possessing functions to modulate gene splicing, transcriptional repression, and RNA metabolism are implicated in the inclusion bodies found within the cytoplasm and nucleus in NDDs. The presence of abnormal TDP-43 can be found in PD, ALS, and Argyrophilic grain disease (AGD) [31, 35].

## 3 ROLE OF NATURAL PRODUCTS IN NDDs

Despite a long history of conventional use and safety credence for natural products, they have woefully received less attention than chemically synthesized drugs [36]. Neurohealth-promoting properties of natural products ranging from improving cognitive function to alleviating NDD’s symptoms including depression, memory loss, and poor cognition are being practiced by traditional medicine practitioners [37-39]. Studies have reported the use of various plant and animal species as pro-cognitive agents and treatment options for several neurological and psychological disorders [40-42]. Considering the ability to modulate the other drug molecular activities, different drugs are provided in combination as per the disease complexity [38].

In the recent decade, natural products have been intensively explored under the spotlight for the discovery and development of novel and effective drug products. The multitargeted nature of phytochemicals at the molecular level makes them perfect to be developed and employed in therapeutics [43]. Potent disease-modifying and -treating actions of phytochemicals have been scientifically established for several NDDs including PD [44], AD [38], ALS [45], and other serious NDDs. Natural products have been a fertile source for the discovery and development of novel therapeutics. Natural products from various classes have been discovered for the potential to treat NDDs, and an update on the compilation is covered further. Fig. (**1**) schematically describes the targets *via* which natural products have been noted to act in order to prevent and treat NDDs.

### 3.1 Alkaloids

These are generally nitrogen-containing, basic organic compounds having mostly complex cyclic structures and the property of being alkali-like. Various alkaloidal entities such as donepezil, galanthamine, caffeine, memantine, and berberine have been scientifically proven to possess the potential for the treatment of NDDs. Fig. (**2**) includes the chemical structures of neuroprotective alkaloids.

#### 3.1.1 Apomorphine

Lipophilicity and affinity of apomorphine for dopaminergic receptors are attributed to the tetracycline ring present in its structure. The efficacy of apomorphine and levodopa are noted to be almost overlapping [46]. The bioavailability of apomorphine is very low due to extensive hepatic first-pass metabolism. Administration of apomorphine has been reported to relieve symptoms of PD and reduce the intermittent “off” periods. Contrastingly, antagonistic activity for serotonergic (5HT_2_A, 5HT_2_B, 5HT_2_C) and adrenergic (α2A, α2B, α2C) receptors has been noted [47]. Additionally, subcutaneous administration of apomorphine has prevented PD-associated motor impairment. It is noted to have better symptom controllability than other long-acting dopamine agonists [48].

#### 3.1.2 Berberine

It is an isoquinoline alkaloid obtained from Chinese medicinal plants including berberis, Hydrastis Canadensis, and Coptis Chinensis. Berberine is noted for its significant neuroprotective role mainly for AD, PD, and HD. It significantly improves AD cases by improving neurotransmission, inhibiting senile plaque and NFTs formation, and inhibiting Tau hyperphosphorylation [49, 50]. Mechanisms behind these significant activities are the inhibitory potential of berberine for cholinesterase (AChE and BuChE), β-site amyloid precursor protein cleaving enzyme 1 (BACE1) [51, 52], Glycogen synthase kinase 3 (GSK-3), oxidants, and inflammation [50].

In PD, the onset of disease or symptoms is alleviated by mainly restoring the dopamine levels and dopaminergic neurotransmission in the brain. Monoamine oxidase enzyme B (MAO-B) is a mitochondrial enzyme that catalyzes the oxidative deamination of monoaminergic neurotransmitters such as Dopamine and 5-Hydroxytryptamine (5-HT) [53, 54]. Past studies have revealed the MAO-B inhibitory activity of berberine which leads to its neuroprotective function in PD [50]. Moreover, docking results have shown that synthetic derivatives obtained from berberine in recent years also possessed significant MAO-B inhibiting properties [55]. Additionally, the neurotoxin-preventive potential has also been justified by researchers [56].

Mutated huntingtin gene and resulting polyglutamine tract are mainly responsible for the development of HD. Induction of autophagy-lysosomal pathway (whose impairment is known to cause HD [57]) is a lead strategy for preventing the development of HD. Autophagy triggering activity of berberine could lead to retardation towards HD progression [58, 59]. Besides, the anti-neurotoxic, anti-neuroinflammatory, and anti-convulsion properties of berberine have been well identified [60].

#### 3.1.3 Caffeine

Epidemiological investigations and animal evidence firmly support that the widely consumed psychoactive substance caffeine possesses beneficial properties for cognitive and motor improvement in PD. Caffeine, by abrogating lipopolysaccharide-induced oxidative stress and neuroinflammation [61], inhibiting adenosine A2A receptor activation, reducing tau hyperphosphorylation, and accumulation of misfolded proteins is reported to provide beneficial effects in PD and AD cases [62]. Interestingly, epidemiological and preclinical studies have shown a lower risk of AD and PD with moderate caffeine consumption (3-5 mg/kg) [63].

#### 3.1.4 Avicine

It is a benzo phenanthridine alkaloid obtained from the roots of *Zanthoxylum rigidum*. Plazas *et al.*, [64] have reported the highly potent reversible-mixed inhibitory activity of avicine and nitidine against acetylcholinesterase enzymes (AChE and BChE). Also, the compounds were found to moderately inhibit MAO-A (IC_50_ = 0.41 ± 0.02 µM) enzyme and Aβ_1-42_ (IC_50_ = 5.56 ± 0.94 µM) aggregation warranting the therapeutic possibilities of avicine for AD treatment.

#### 3.1.5 Harmine

Harmine is a β-carboline alkaloid abundantly found in *Peganus harmala* L. Studies have attributed the pharmacological anti-Alzheimer activity of harmine to its inhibitory activity for AChE, oxidation, and inflammation. Harmine decreased the brain levels of GABA which is implicated in many neurological diseases. Also, it was reported to inhibit AChE in rat endothelial brain (REB) cells [65]. Harmaline, an analog to harmine was found to be more effective (2 mg/kg) than harmine (20 mg/kg) at a relatively lower dose [66].

The antiparkinsonian effects of harmine comparable to levodopa have been documented as well. The water-soluble form of harmine *i.e*. harmine hydrochloride was found to have anti-cataleptogenic (haloperidol-induced catalepsy) effect at doses of 2.5 and 5 mg/kg. Additionally, harmine at the same doses reduced oligokinesia induced by the administration of 30 mg/kg 1-methyl-4-phenyl-1,2,3,6-tetrahydropyridine (MPTP) neurotoxin [67]. Furthermore, harmine was found to time- and dose-dependently degrade different forms of α-synuclein. It upregulated PSMD1 subunit of the ubiquitin-proteasome system to promote α-synuclein degradation by enhancing PKA phosphorylation [68].

#### 3.1.6 Huperzine A

It is a promising anti-Alzheimer alkaloid of sesquiterpene class extracted from firmoss *Huperzia serrata* having potent, selective, and reversibly inhibiting properties for the AChE enzyme. The AChE inhibiting activity of huperzine A is more potent than donepezil, galantamine, and rivastigmine [69]. Huperzine A inhibits AChE by regulating the accumulation of Aβ accumulation [70]. Microwave-assisted *H. serrata* green extract prevented cortical neurons exposed to glutamate from neurotoxicity and hence increased neuronal survival. Interestingly, two phenolic acids, caffeic acid, and ferulic acid were found to synergize the neuroprotective effects of huperzine A *via* the extracellular-signal-regulated kinase (ERK) pathway [71]. Moreover, huperzine is known to inhibit transferrin receptor protein 1 (TFR1) whose upregulation is implicated in increased iron uptake by neuronal cells and the development of AD [70].

#### 3.1.7 Piperine

The pharmacological effects of piperine on the central nervous system including anti-convulsant, antidepressant, and anti-ischaemic are already known. Additionally, the anti-Alzheimer activity of piperine has been corroborated by recent studies. Piperine prevented altered neurotransmission, oxidative-nitrosative stress, and hippocampal neurotransmission in intracerebroventricularly infused streptozocin-induced Alzheimer’s disease in experimental mouse models [72]. The results from preclinical studies suggest that piperine easily crosses BBB and is thus effective for neurological disorders such as AD, PD, and HD [73]. Piperine has been noticed to promote autophagy flux in SNCA overexpression-induced PD cell and mouse models. PIP treatment was found to attenuate olfactory and motor deficits accompanied by degradation of pathological SNCA in the olfactory bulb, an accumulation of which is one of the earliest non-motor symptoms of PD [74]. In addition to the above, the combination of piperine with quercetin has been experimentally noted to be effective in treating iron supplement- and rotenone-induced PD in rats [75].

Additionally, Salman *et al.*, concluded the anti-huntington activity of piperine against 3-nitropropionic acid (3NP)-induced neuropathology in animals. Piperine treatment reduced neuronal loss and astrocytes activation in the striatal brain region. Also, piperine prevented and alleviated biochemical, behavioral, immunohistochemical, and histological alterations in 3-nitropropionic acid administered male Wistar rats [76].

#### 3.1.8 Capsaicin

Capsaicin (8-Methyl-N-vanillyl-6-nonenamide), a major alkaloid found in capsicums has been repeatedly noted to be effective against several neurological-related proteinopathies including chemical-induced AD and PD [77]. In recent studies, capsaicin has been noted to alleviate AD-related pathologies including neuro-inflammation and degradation, and tau hyperphosphorylation. Aβ burden was found to be reduced in APP/PS1 mice by switching amyloid precursor protein towards α cleavage [78]. Additionally, capsaicin treatment has declined the amount of tau and Aβ in the hippocampal homogenate of STZ-induced AD rat models [79].

In addition to the above, researchers have concluded that capsaicin exerts an apoptosis-alleviation effect in the 6-OHDA-induced PD cell model by regulation of apoptosis-causing genes *viz*. Actg1 and Gsta2 [80]. In a study, researchers determined the radical and superoxide anion reducing the potential of capsaicin in PD model flies. Capsaicin was found to alleviate lipid peroxidation (LPO) (by 1.8 fold), glutathione-S-transferase (GST) (by 1.26 fold), and MAO activity (by 1.6 fold) while significantly increasing dopamine (1.56 fold) and glutathione (by 1.37 fold) content [81].

### 3.2 Flavonoids

Flavonoids are polyphenolic secondary plant metabolites, commonly found in fruits and vegetables in the form of aglycones or glycosides. Possessing a common skeleton of phenyl-benzopyran (C6-C3-C6), they are classified into several categories including flavones, flavonols, flavanones, isoflavones, flavonoids, and anthocyanidins. These are among the most intensively explored phyto-secondary metabolites owing to indispensable health benefits [82-84]. Various flavonoids such as naringenin, nobiletin [85], cocoa [86], fisetin, and apigenin have been noted to show significant antioxidant and neuroprotective functions. Fig. (**3**) presents the chemical structures of neuroprotective flavonoids.

#### 3.2.1 Naringenin

The compound has already been reported to be neuroprotective due to its antioxidative and anti-inflammatory properties [87, 88]. In a study, the effect of naringenin nanoemulsion against Aβ-induced neurotoxicity in human neuroblastoma cell lines (SH-SY5Y) was determined. Naringenin alleviated direct neurotoxicity caused by Aβ by reducing amyloidogenesis indicated by the downregulation of BACE and APP expression. Furthermore, the levels of tau phosphorylated proteins were reduced [89]. Additionally, naringenin has been noted to favorably and strongly bind to collapsing response mediator protein-2 (CRMP 2), a neurotherapeutics target, and disrupts kinase-mediated phosphorylation [90]. Recently, various naringenin derivatives possessing anti-AD properties have been designed and synthesized [91, 92].

Paraquat is an oxidative stress-causing herbicide and is implicated in the development of PD. Studies have reported that naringenin inhibits or modulates paraquat-induced RNA expression of SNCA, DAT, β-catenin, caspase-3, and BDNF genes, and hence exhibits neuroprotection against paraquat-induced neurodegeneration [93]. Naringenin has improved behavioral response and neurotoxicity caused by several PD-causing chemical agents including MPTP [94], 6-OHDA [95], and LPS [96]. Moreover, the binding and inhibitory potential of naringenin (-12.0 kcal/mol) for the active site of MAO-B has also been reported [97].

In addition to the above, the neuroprotective effects of naringenin against 3-NP-induced Huntington-like symptoms have also been reported. Naringenin administration caused a reduction in the glial fibrillary acidic protein (GFAP) and attenuation of neuronal cell death in Wistar albino rats [98].

#### 3.2.2 Apigenin

The neuroprotective actions of apigenin have been well explored by scientists across the globe. Apigenin has been found to possess neuroprotective properties in AD-associated inflammation by reducing microglial activation, modulation of expression of inflammatory cytokines, and increasing the expression of brain-derived neurotrophic factor (BDNF) *in-vitro* [99]. The expression of glycogen synthase kinase -3 (GSK-3), a critical molecule modulating major hallmarks of AD, is reported to be decreased on apigenin (50 mg/kg) administration resulting in suppressed BACE1 expression and reduced tau hyperphosphorylation [100]. Besides, apigenin has been noted to provide neuroprotection against acrylonitrile by inhibiting TLR4/NF-κB signaling pathway and neuron apoptosis [101].

The anti-PD potential of apigenin has also been investigated in recent years. In a study, the apigenin was found to prevent neuroinflammation in substantia niagra pars compacta, attenuated NF-κB gene expression, and inhibited the rotenone-induced release of proinflammatory cytokines IL-6 and TNF-α. Additionally, apigenin prevented the reduction in mRNA expression of GDNF and BDNF in rats [102].

In ethidium bromide-induced multiple sclerosis experimental rat models, apigenin prevented neurochemical and neurobehavioural defects by modulating the levels of c-JNK/p38MAPK and myelin basic protein in brain and CSF homogenate. Moreover, the imbalanced levels of apoptotic markers (Bax, Bcl-2, caspase-3) and inflammatory cytokines (TNF-α, IL-1β) were reduced by apigenin treatment [39]. Also, the oxidative stress amelioration potential of apigenin in SH-SY5Y [103] and dendritic cells of experimental autoimmune encephalomyelitis (EAE) mice [104] has been documented.

#### 3.2.3 Luteolin

Luteolin (5,7,3',4'-Tetrahydroxyflavone) is a flavone type of flavonoid mainly found in Reseda luteola. The anti-AD potential of luteolin has been largely supported by its ER stress suppression [105] and anti-neuroinflammatory actions for IL-1β, IL-6, TNF-α, COX-2, NO, and iNOS. In a triple transgenic mouse model of AD, luteolin (1 to 10 µM) improved spatial learning and ameliorated memory defects along with inhibited LPS-induced cell proliferation, ER stress marker GRP78 increase, and excessive cytokine release in astrocytes [106]. In another study on transgenic fly lines having wild-type human Aβ42, luteolin dose-dependently reduced AChE, catalase (CAT), GST, LPO, PCC, and caspase-3 and caspase-9 activity. Additionally, luteolin reduced the expression of Aβ_42_ expression dose-dependently [107].

In several PD models, luteolin has been reported to act by inhibiting the release of and/or decreasing the levels and activity of lactate dehydrogenase, caspase-3, IL-1β, TNF-α, and reducing mRNA levels of Lrrk2 [108, 109]. Interestingly, the adjuvant therapy value of palmitoylethanolamide/luteolin to treat dyskinesia and camptocormia has been reported [110].

Besides, luteolin is reported to interact with the mutant form of HTT. Administration of luteolin to transgenic drosophila HD model flies resulted in a dose-dependent decrease in oxidative stress and delay in loss of climbing activity. This suggested the effectiveness of luteolin to reduce symptoms of HD [111].

#### 3.2.4 Quercetin

Quercetin (pentahydroxyflavone), having a bitter flavor is commonly found in capers, red onions, and kale. In addition to its antioxidant and anti-inflammatory activities, other activities including hypolipidemic, hypoglycemic, activities have been intensively explored and documented [112, 113]. The molecular mechanisms behind the neuroprotective effects of quercetin in AD chiefly include the potentiality to down- or up-regulation of cytokines *via* protein kinase c (PKc), c-Jun N-terminal kinase (JNK), nuclear factor (Nrf2), paraoxonase-2, and mitogen-activated protein kinase (MAPK) signaling cascades and alteration is antioxidant enzymes (catalase, GST) [114-116]. Additionally, the derivative, quercetin-*O*-glucuronide has been noted to alleviate Aβ_1-42_cognitive toxicity by modulating the gut-brain axis in AD mice models [117]. In a recent study, quercetin (25 and 50 mg/kg) has been noted to up-regulate the gene expression of ADAM-10 and ADAM-17 GENES to prevent neurotransmission impairment and cognitive deficits in AlCl3-induced AD rats [118].

In rotenone-induced PD animal models, quercetin (50 mg/kg p.o) supplementation improved neurotransmitter levels by increasing the levels of antioxidant enzymes and hence improved cognitive and motor functions and reduced behavioral depression [119]. Additionally, quercetin-rich extract rich from Chrysobalanus icaco has shown potent NADP oxidase inhibition (IC_50_ = 8.1 μg/mL) with a docking score of ΔG = -8.3 kcal/mol on NADPH oxidase [120]. Also, quercetin has shown neuroprotective effects in 6-OHDA-lesioned rat PD models by reducing mitochondrial damage and α-synuclein accumulation and mitigating neuronal death *in vivo* [121]. Interestingly, the nanoparticle formulation of quercetin has shown the potential to inhibit mHTT aggregation in the HD cell model [122].

#### 3.2.5 Fisetin

Fisetin (3,3′,4′,7-Tetrahydroxyflavone), a potent antioxidant flavone compound is mainly found in strawberries, apples, onions, and parrot trees. Fisetin has shown inhibitory potential toward the aggregation of tau fragment K18. Fisetin directly interacts and strongly binds with tau K18 protein, prevents the β-strand formation at critical hexapeptide motifs, and prevents tau aggregation [123].

In rotenone-induced rat PD models, fisetin (10 to 20 mg/kg p.o.) reversed rotenone-induced mitochondrial enzyme changes, striatal dopamine levels, and normalized antioxidant enzyme levels [124]. In another study, fisetin significantly suppressed 6-OHDA-mediated activation and elevation of caspase-3, caspase-9, and expression of oxidative stress-related genes in human neuronal SH-SY5Y cells by activating PI_3_K-Akt signaling [125]. Besides, fisetin has been noted to prevent degeneration in PD by inhibiting protein aggregation, mitochondrial dysfunction, glutathione loss, and inflammatory conditions [126]. Furthermore, fisetin has been noted to inhibit α-syn fibrillation by binding with- and providing structural rigidity to α-syn and hence delaying the onset of structural transitions [127].

In multiple hSOD-1 mutated Drosophila models of ALS, fisetin has lessened both mutant and wild hSOD-1 *in vivo* and *in vitro* and has shown significant neuroprotection by attenuating motor impairments, regulating redox homeostasis, and reducing ROS damage [128].

#### 3.2.6 Hesperidin

Hesperidin is a flavanone glycoside majorly present in fruits (sweet oranges), vegetables (lemon), and polyherbal formulations. Its potential to suppress oxidation and inflammation contributes to cardioprotective, antihyperlipidemic, antidiabetic, and antihypertensive properties. For neuroprotection in AD, hesperidin has been noted to act by attenuating inflammation, caspase activation, raised p-Tau levels, and Aβ-42 and AchE overactivity in AlCl3-intoxicated rats [129, 130], and scopolamine-induced AD-like rat model [131].

In PD, hesperidin has shown significant neuroprotective effects by intracellular calcium homeostasis, restoring dopamine levels, improved motor function, ameliorating oxidative stress and mitochondrial dysfunction, downregulating gsk3β, lrrk2, polg, and casp9 genes in 6-OHDA-toxicated SH-SY5Y cellular [132], and zebrafish model of PD [133], and iron-induced Drosophila model of PD [134].

#### 3.2.7 Curcumin

Curcumin is an indispensable compound commonly obtained from the rhizomes of *Curcuma longa*. The intensive exploration of the molecule across the globe has revealed its significant valuable properties to be employed in the therapeutics of cancer, NDDs, infectious diseases, and metabolic syndrome. The neuroprotective effects of curcumin against AD, PD, MS, and cerebral ischaemia have been experimentally validated that evinces the pleiotropic effects of curcumin including antiapoptotic, anti-inflammatory, antioxidative, and vasculo-protective effects [135]. Mechanisms underlying its anti-AD effects include but are not limited to preventing antioxidant enzyme activity, increasing the synthesis of BDNF, reducing the levels of ROS, TNF-α, IL-1β, IL-6, malondialdehyde (MDA) [136, 137], and affecting the aggregation of tau and amyloid proteins [138, 139]. Recently, the neuroprotective effects of curcumin have been noted for several AD models such as AlCl3-induced- [140], scopolamine-induced- [137], and streptozotocin-induced rat AD model [141]. In experimental *in-vitro* and *in-vivo* AD models, curcumin has been shown to reverse neurotoxic and behavioral damages which warrants its administration as a promising approach [142].

Interestingly, the inducing effect of curcumin on IL-10, a potent anti-inflammatory, and immunosuppressive cytokine is an important mechanism behind its anti-PD effects [143]. Curcumin modulates the immune system *via* the cholinergic anti-inflammatory pathway by selectively stimulating α7 nicotinic acetylcholine receptors (α7-nAChR), which is a novel proposed approach to treat PD [144]. Additionally, the neuroprotective effects of curcumin for paraquat-induced PD model fibroblasts [145], rotenone-induced PD model [146], 6-OHDA-lesioned rat PD model, and PC12 cells [147] prevent mitochondrial dysfunction, alleviating oxidative stress, inhibiting AKT/mTOR signaling pathway, and reducing lipoperoxidation [148]. Furthermore, the binding of curcumin to non-amyloid β-component of α-synuclein has been hypothesized to prevent the spread of- and clarifies α-synuclein from the body [149].

In the R6/2 mice model of HD, a curcumin-supplemented diet protected from neuropathology and GI dysfunction maintained normal motor function, and conserved intestinal contractility [150]. Besides, curcumin effectively mitigated HD symptoms including metabolic derangements and ROS levels in the Drosophilia model of HD [151]. Surprisingly, curcumin supplementation has shown retardation in disease progression towards ALS and improved aerobic metabolism in a trial that prophesies its possible positive effects on ALS [152].

#### 3.2.8 Rutin

It is citrus flavonoid glycoside between quercetin and rutoside, commonly found in vegetables, herbs, and fruits such as asparagus, tobacco, viola, buckwheat, and forsythia. In addition to hypolipidemic, anti-hypertensive, anti-allergic, anti-bacterial, antiprotozoal, and antitumor properties, the neuroprotective effects of rutin are well known [153]. Recently reported mechanisms underlying the anti-AD effect of rutin include regulation of tau hyperphosphorylation, inhibition of tau aggregation and tau oligomer-induced toxicity, and suppression of neuroinflammation by downregulating NF-κB pathway, and promotion of microglial uptake of extracellular tau oligomers [154]. Recently, Ouyang *et al.* developed a nanoflower formulation comprising rutin and microRNA-124. Rutin was predicted to synergize microRNA-124, which could suppress the expression of APP and BACE1 in the hippocampus of AAP/PS1 mice [155].

The anti-HD effects of rutin are too well investigated. In Caenorhabditis elegans model of HD, rutin, by controlling the expression of antioxidant enzymes, activation of protein degradation and insulin/IGF1 signaling pathways, maintained the ASH neuronal function, reduced the degeneration of their sensory terminations, and reduced polyglutamine protein aggregation [156, 157]. Also, rutin alone (25 and 50 mg/kg), and rutin along with selenium has shown significant anti-HD effects in a 3-NP-induced rat and mice model of HD evinced by restoration of biochemical, behavioral, and histological alterations which were further caused due to inhibiting astrocyte activation, increasing BDNF, and improving cholinergic and monoaminergic transmission [158, 159].

#### 3.2.9 Mucuna

*Mucuna pruriens*, belonging to the Fabaceae family consists of phenolic components which execute a significant neuroprotective effect in humans. It consists of dopamine precursor L-DOPA, which is the reason for its employment in treating PD cases in Ayurvedic medicine. Additionally, the plant is used in the treatment of depressive neurosis and snakebites. Studies have shown the preventive effects of *M. pruriens* against neurological damage caused by bilateral carotid artery occlusion-induced global cerebral ischaemia in rats that warrants its adjuvant use to improve the life quality of PD patients [160, 161].

### 3.3 Terpenoids and Carotenoids

Terpenoids or isoprenoids are the largest class of plant secondary metabolites. A wide array of pharmacological properties including anticancer, antiprotozoal, antioxidant and anti-inflammatory, and neuroprotective properties have been confirmed by *in-vitro*, preclinical, and clinical studies. Based on the number of forming isoprene units, terpenoids are classified into monoterpenoids (C10), sesquiterpenoids (C15), and diterpenoids (C20), sesterterpenoids (C25), triterpenoids (C30), tetraterpenoids (C40) [162]. The neuroprotective effects of different terpenoids are covered below. The chemical structures of investigated terpenoids and carotenoids possessing neuroprotective activities are given in Fig. (**4**).

#### 3.3.1 Crocin

Crocin (crocetin di-gentibiose ester) is a water-soluble carotenoid found in crocus and gardenia flowers. A broad range of pharmacological effects on the cardiovascular system, liver disease, joint disease, neurodegeneration, diabetes, depression, and tumor have been investigated [163]. In various AD models, crocin has caused significant alleviation of disease progression and improvement by different mechanisms including blocking Aβ-initiated apoptosis [164], preventing mitochondrial damage [165], attenuating expression of TNF-α and IL-1β mRNA [166], reducing Aβ deposition in the brain hippocampus, and reducing ROS generation *via* its anti-inflammatory effects [166, 167].

Additionally, crocin in doses of 10, 20, and 40mg has shown protective effects towards malathion-induced PD-like behavior in rats through anti-inflammatory effects and reducing lipoperoxidation as evinced by increased levels of GSH and decreased MDA, TNF-α, and IL-6 levels in the striatum [168].

Interestingly, the spectrum of neuroprotective effects of crocin is further extended toward HD. In *Drosphilia melanogaster* HD flies, crocin has been reported to prevent Escherichia coli colonization, which is implicated in HTT aggregation and HD-associated immobility [169].

#### 3.3.2 Astaxanthin

Astaxanthin is a strong antioxidant, marine-derived ketocarotenoid, generally known as beneficial for skin problems including aging, dehydration, and wrinkles [170]. Astaxanthin could impede the development of AD by inhibiting aggregation of Aβ_1-42_ and hence its toxicity in PC12 cells. Although astaxanthin possesses the highest bioavailability of other carotenoids (lutein and carotene), the bioavailability can be further enhanced by carrier oils such as polysorbate 80 [171]. In Aβ_1-42_ in toxicated Wistar rat models of AD, astaxanthin dose-dependently and significantly reversed the cognitive and memory impairment that was evinced by reduced levels of Aβ_1-42_, AChE, TNF-α, oxidative stress, GSK-3β, and IRS-S307 activity in the hippocampus [172]. Sakayanathan *et al.* have noted that astaxanthin-s-allyl cysteine inhibited human brain AChE (IC_50_ = 0.80 µM) and human serum BChE (IC_50_ = 0.800 µM) by interacting with amino acids present in peripheral anionic and cationic site of both enzymes [173]. Being widely employed following haemorrhagic stroke, *Astralgus membranaceus*, by its antioxidant and anti-inflammatory action, has been noted to enhance functional recovery of the patient if treatment is started within 24 hours [174, 175].

Surprisingly, the neuroprotective effects of astaxanthin on PD have been well extended. In 1-methyl-4-phenylpyridinium (MPP+) caused PD in SH-SY5Y cells, astaxanthin, by targeting miR-7/SNCA axis, inhibited endoplasmic reticulum stress and hence abolished the promotion of apoptosis and inhibition of cell viability [176]. Similar actions were reported in rotenone-intoxicated *Drosophilia melanogaster* models of PD. Trans-astaxanthin (0.5 and 1.0 mg/10 g diet) inhibited the rotenone-mediated decrease of thiol content, inhibition of catalase, acetylcholinesterase, and glutathione-s-transferase activity. Additionally, the docking score of trans-astaxanthin against pro-inflammatory targets was higher than standard inhibitors [177]. In addition to the above, astragaloside IV and tetramethylpyrazine, extensively used medicines for the treatment of myocardial ischaemic conditions, the combination has shown a synergistic protective effect against cerebral ischaemic-reperfusion injury by downregulating MDA, iNOS, caspase-3, and upregulating SOD, and Bcl-2 [178].

#### 3.3.3 Lutein

Lutein, a structural isomer of zeaxanthin is a non-provitamin A dietary carotenoid noted to have strong anti-oxidant properties in experimental studies. Having a property to preferentially accumulate in macula lutea to protect the retina from UVR-caused oxidative damage, lutein also possesses anti-inflammatory, anticarcinogenic, and skin-friendly properties [179]. In Aβ_25-35_-intoxicated rat PC12 cells, lutein extract from Bombyx mori yellow cocoons significantly ameliorated toxicity by attenuating ROS production, MAPKs pathway activation, apoptosis, and loss of cell viability [180]. In another study, lutein (0.1-1 µM) prevented SH-SY5Y cells from OxPL 1-palmitoyl-2-(5'-oxo-valeroyl)-sn-glycero-3-phosphocholine (POVPC) toxicity indicated by decreased ROS production and prevention of loss of GSH [181].

Fernandes *et al.* have investigated the anti-PD effects of lutein-loaded nanoparticles in *Drosphilia melanogaster* models of PD. Lutein executed anti-PD effects by restoring the levels of dopamine, tyrosine hydroxylase, oxidative stress indicators, and AchE activity and hence preventing PD flies from rotenone-induced locomotor damage [182].

Although a very small search has been performed to investigate the anti-HD effects of lutein. Lutein 50 or 100 mg/kg orally (p.o.) has shown considerable anti-HD effects in 3-NP intoxicated rat models of HD by normalizing body weight, improving mitochondrial enzyme complexes, and attenuating oxidative stress [183].

#### 3.3.4 Crocetin

It is an amphiphilic apocarotenoid dicarboxylic acid and the chief active component of saffron responsible for a broad range of pharmacological properties. It is known to be a potential autophagy inducer in AD. In N9 microglial and primary neuron cells, crocetin induced autophagy by activating STK11/LKB1 (serine/threonine kinase 11)-mediated AMP-activated protein kinase (AMPK) pathway and hence resulted in clearance of Aβ. Additionally, APMK-activation has resulted in Aβ lowering in transgenic male 5XFAD mice models of AD [184]. In another study, crocetin has significantly reduced pro-inflammatory cytokines, suppressed NF-κB activation and p53 expression in mice hippocampus, decreased levels of Aβ, and enhanced anti-inflammatory cytokines [185].

In MPTP-induced PD model animals, crocetin has exerted excellent antioxidant and anti-inflammatory effects both *in vivo* and *in vitro*. It prevented animals from MPP+ induced mitochondrial damage by regulating mitochondrial permeability transition pore (MPTP) viability and decreasing the expression of inflammatory and proinflammatory cytokines [186].

#### 3.3.5 Beta Carotene

Beta carotene is a red-orange-colored pigment and precursor of vitamin A. Having three isomers of beta carotene *i.e*. alpha, beta, and gamma, the beta isomer is the most active. The natural compound is known generally for its strong antioxidant properties [187]. To investigate its effectiveness for neuroprotective effects against AD, Hira *et al.* injected beta carotene in 50 streptozotocin (STZ)-intoxicated male albino mice models of PD. Beta carotene (2.05 mg/kg), by exerting strong antioxidant effects, inhibiting AchE, and reducing Aβ-protein fragments attenuated STZ-induced memory deficit. Furthermore, *in silico* studies revealed the considerable binding capacity of beta carotene with the AchE enzyme [188].

It has been noted that PD patients have low serum levels of beta-carotene and lycopene, which may predispose them to oxidative stress and hence the development of PD [189]. Further research to investigate mechanistic pathways of beta carotene to treat or prevent PD is needed.

#### 3.3.6 Lycopene

Lycopene is a naturally occurring and red-color contributing carotenoid pigment mainly found in tomatoes, papaya, and watermelons. Other than neuroprotection, the strong antioxidant action of lycopene contributes to the antiapoptotic, antimetastatic, and antiproliferative actions of lycopene [190]. Huang and coworkers reported that lycopene possesses the potential to prevent the development of oxidative stress-mediated AD. In cerebrocortical neurons of mouse models of AD, lycopene enhanced cell viability, increased GSH/GSSG levels, decreased production of ROS, and restored mitochondrial membrane potential. Additionally, lycopene increased synaptophysin levels, activated PI_3_K/Akt pathway, and reduced the ratio of cleaved caspase 3: caspase 3 which signifies its efficacious anti-AD potential [191].

Lycopene has been concluded by authors to have PD-modifying properties. It possibly acts by reducing the loss of dopaminergic neurons, showing an anti-amyloidogenic effect, and reducing ischaemic-related apoptosis [192].

#### 3.3.7 Fucoxanthin

Fucoxanthin is a potent antioxidant allenic carotenoid that is isolated mainly from marine sources such as macroalgae and microalgae. It structurally resembles dinoxanthin and neoxanthin. After being administered orally, fucoxanthin is metabolized into fucoxanthinol and amarouciaxanthin A, which are accumulated in the heart and adipose tissues, respectively. Fucoxanthin is found to be a potential candidate against obesity, diabetes, cancer, and heart and liver diseases [193, 194].

Being a potent antioxidant and anti-inflammatory compound fucoxanthin is used in various neurological disorders such as PD, AD, and TBI. It is also known to inhibit the production of proinflammatory cytokines (TNF-a, IL-1b, and IL-6), cyclooxygenase-2 (COX-2), and nitric oxide (NO). Fucoxanthin has been noted to have hydrophobic interactions with Aβ peptides. Fucoxanthin, by interacting with Aβ prevents the conformational transition and Aβ oligomer formation. It also increases the expression of BDNF and significantly inhibits oxidative stress [195]. Additionally, fucoxanthin has been noted to inhibit LPS/ATP-induced secretion of pro-inflammatory cytokines. Fucoxanthin reduced the protein expression of ASC, NLRP3, and cleaved caspase-1 to inhibit NLRP3 inflammasome and NF-κB activation [196].

Targeting oxidative stress to be the major culprit for neuronal damage in PD, Wu *et al.* investigated the effect of fucoxanthin in 6-OHDA stressed PC12 cells. Results suggested that fucoxanthin acts to prevent apoptosis by targeting Keap 1 (Kelch-like ECH-associated protein 1), which is a suppressor of Nrf2 to prevent the interaction between Keap 1 and Nrf2. Besides, fucoxanthin suppressed 6-OHDA induced disruption of mitochondrial membrane potential, and intracellular ROS accumulation [197].

## 4 SUCCESS STORIES

The complex nature and multifactorial development of NDDs, ADRs of chemically synthesized drugs, and the development of resistance among patients have propelled scientific interest towards natural and therefrom derived molecules for novel drug development [198]. Numerous molecules have been synthesized by modifying naturally occurring molecules have been found to exhibit significant neuroprotective properties. Fig. (**5**) composes examples of hybrid molecules synthesized and possessing neuroprotective properties.

The exploration of natural resources including plants, minerals, and marine sources has been on trend for the past decades. The naturally occurring molecules belonging to different pharmacognostic classes have been reconnoitered intensively as evinced by accumulated literature and scientific reports. This has emerged with numerous novel and effective natural product formulations to treat NDDs. Additionally, efforts have been made to address the pharmacokinetic issues. In an attempt to enhance the biopharmaceutical and bioavailability characteristics of natural products, formulations including nanoparticles, nanocarriers, inclusion complexes, and self nano-emulsifying drug delivery systems (SNEDDS) have been developed. Table **1** [199-217] depicts the recently developed natural product formulations for the prevention and treatment of NDDs.

Additional to the above, successful research studies have continued the natural products and their formulation up to clinical trials and patents too which evinces the success of natural products in drug discovery and development. Regardless of whether it is a carcinogenic, neurological, or metabolic disorder, natural products have grabbed abundant attention for the discovery and development of novel drug molecules [243]. We have used the official website of clinical trials *i.e*. CLINICALTRIALS.GOV for the exploration and compilation of clinical trial studies. Table **2** [218-242] presents the clinical trial studies done on natural products to investigate neuroprotective activities. Inclusion criteria for clinical trial studies included; only completed and active studies, and those done for natural products related to neurological disorders since 2010 were selected [243]. Besides, numerous patents for natural products intended for the prevention, disease modification, or treatment of NDDs have been granted. Some examples of patents granted since 2010 are given in Table **3** [244-253]. For patents, the online database PUBCHEM and LENS.ORG (website) was used.

## 5 CHALLENGES AND ETHICAL ISSUES

Although a vast background of historical use and an abundance of phytochemicals, the operation from discovery to development of natural products up to mercenary has been back-breaking. The featured characteristics of natural products over conventional molecules present both advantages as well as challenges for drug discovery as well as development. Screening of natural products for target-based assays has been so onerous due to the vast libraries of extracts obtained from them. The task of pinpointing novel and serviceable natural product molecules from libraries additionally with precluding existing molecules to avoid the rediscovery of existing molecules is a laborious task [254]. Besides, the procedures of extraction span including several difficulties such as extensive duration of extraction, high energy consumption, small extract recovery, and use of petroleum-derived solvents, which are globally getting depleted gradually [255].

Additionally, collecting abundant starting material to isolate and characterize natural product molecules with biological activity is also challenging. Lack of access to enough samples has also led to difficulty in drug discovery of marine-derived natural products [256]. Besides, the intellectual property (IP) protection of naturally occurring molecules can be arduous work due to the inability to IP-protecting natural products in their existing natural form [257]. In addition, the regulations framed in United Nations 1992 Convention on Biological Diversity and Nagoya Protocol defines the need of sharing benefits with the countries where the biological material originates. This poses an auxiliary layer of complexity over the development and protection of natural products [244, 258]. Furthermore, the water insolubility, photosensitivity, poor bioavailability, BBB crossing, and physical and chemical instabilities make natural products intended for NDDs more challenging to be developed [259].

However, it has been noted and reported by authors that an ethical issue regarding the use of natural products exists regarding the safety and efficacy of the product. This highlights the need to advance the interpretation of the molecular mechanism of action of natural products [260].

## CONCLUSION

As an epilogue, nature presents an abundant and outstanding source of neuroprotective natural products. The ethnobotanical use of natural products for healing several ailments including neurological disorders warrants their use in modern times too. Various classes of Phyto-secondary metabolites including alkaloids, glycosides, terpenoids, flavonoids, and combinations thereof possess significant disease-preventive and -modifying properties that are being investigated to act by crucial mechanisms. A common characteristic mode of preventing or treating NDDs is noted to be the antioxidant potential of natural products. The potent NADPH oxidase inhibitory action of quercetin and myricetin has been attributed to their significant antioxidant action against neurodegeneration as well as other diseases. Besides, the inhibiting effect on the aggregation of NDDs-causing proteins has also prevailed in their mechanism of action.

Although, several issues related to extraction, bioavailability, safety, and efficacy need to be addressed. Recent technological and scientific advances to overcome challenges in natural product drug discovery and development present special emphasis on analytical techniques, genome engineering, and cultivation systems. The number of chemical modification and formulation studies to increase the functional activity, and overall safety and efficacy characteristics have risen in recent years. Besides, the clinical trial data is an essential component needed to warrant and underline the safety of the human use of natural products. On the whole, natural products are indispensable and needed sources for drug discovery and drug development. Also, work to investigate their mechanisms of action, and improve extraction yield, efficacy, and safety is needed.

## Figures and Tables

**Fig. (1):**
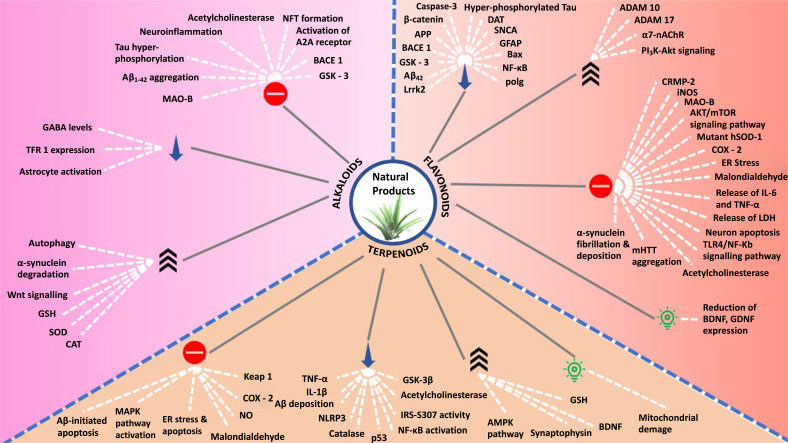
Potential targets for natural products to treat NDDs.

**Fig. (2):**
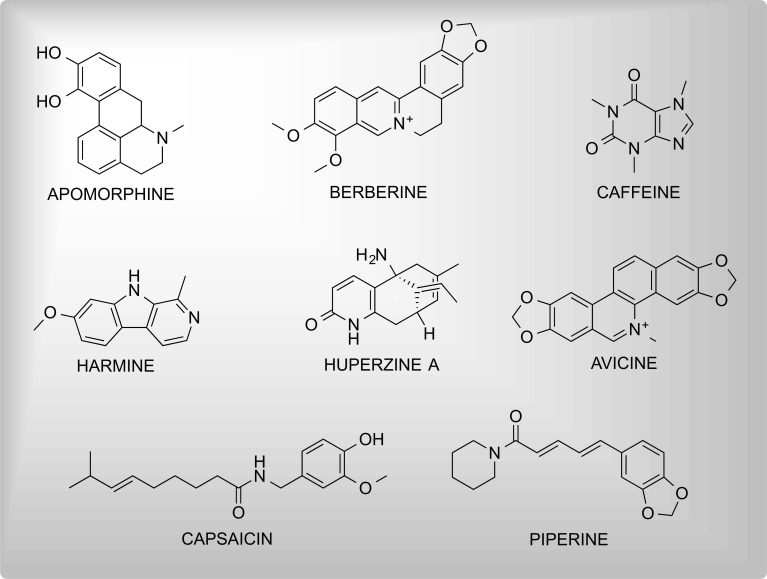
Some significant neuroprotective alkaloids.

**Fig. (3):**
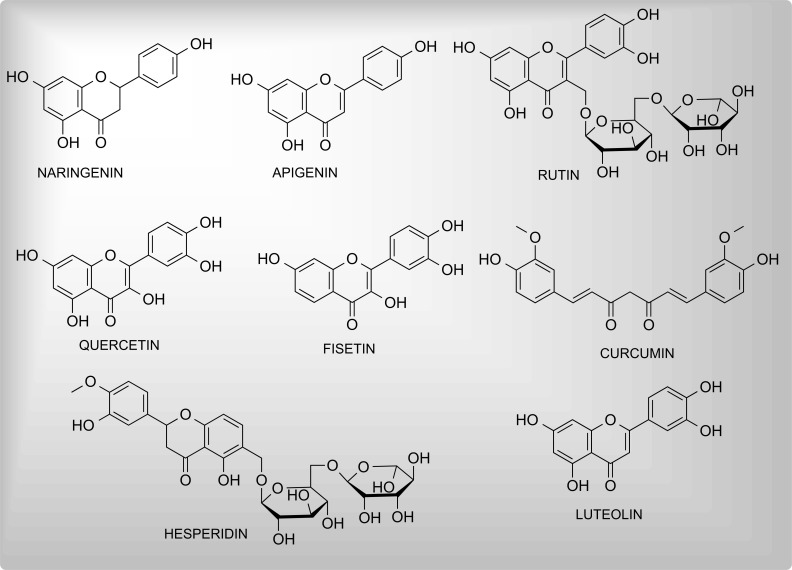
Some significant neuroprotective flavonoids.

**Fig. (4):**
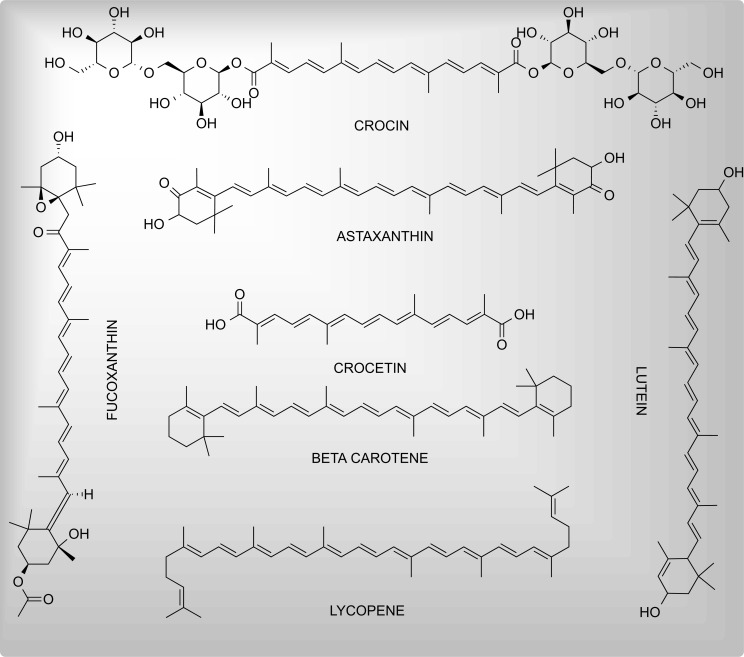
Neuroprotectant carotenoids and terpenoids.

**Fig. (5):**
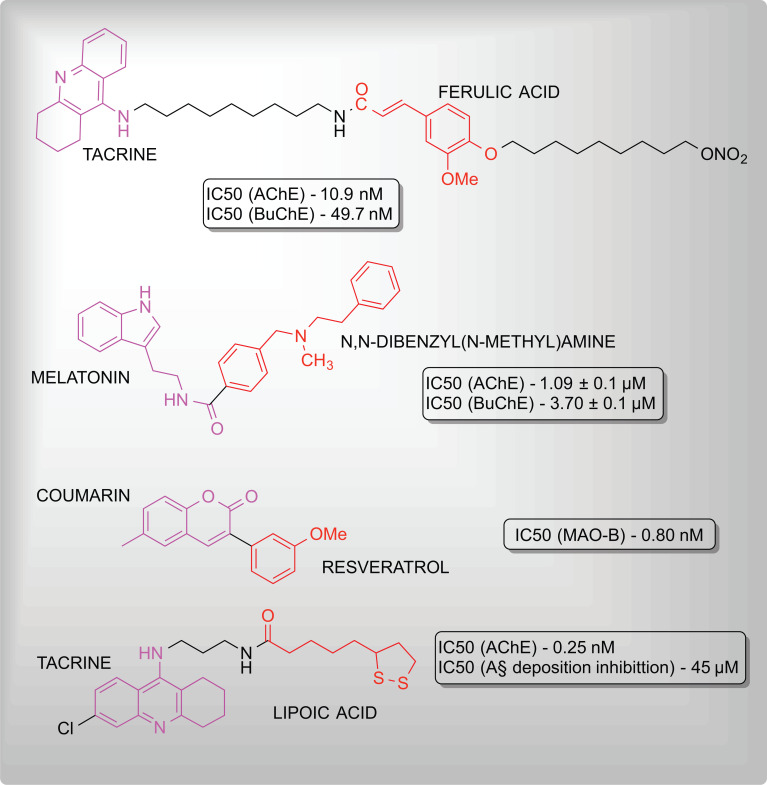
Hybrid molecules with neuroprotectant activities.

**Table 1:** Various formulations of natural products against neurodegenerative disorders.

S. No.	Type of Formulation	Active Natural Components	Formulation	Enhanced Features	Health Advantages	References
1	Nanoparticles	Curcumin	Curcumin encapsulated Pluronic F127 nanoparticles and P68 + DQA curcumin	• Brain accumulation of curcumin following IV dose • Maintenance of Aβ binding property of curcumin • Increased cellular antioxidant activity	Treatment of AD and PD	[199] [200]
		Silibinin	Silibinin-human serum albumin (HSA) nanoparticles	• Increased neuroprotective and antioxidative activity than free silibinin	Treatment of AD	[201]
		Catechin	Trehalose-conjugated, catechin-loaded polylactide nanoparticles	• Enhanced cell proliferation against polyQ aggregates	Treatment of HD	[202]
		Quercetin	Quercetin-loaded nanoparticles	• Antiamyloidogenic effect at lower concentration of quercetin • Improved overall performance than free quercetin	Treatment of HD	[203]
		Epigallocatechin-3-gallate	Epigallocatechin-3-gallate/Ascorbic acid nanoparticles	• More effective to reduce motor defects and depression-like behaviour	Treatment of HD and other neurological disorders	[204]
2	Inclusion complexes	Morin	Morin/hydroxypropyl-β-cyclodextrin inclusion complex	• Improved solubility • Improved solubility and dissolution rate	Anti-inflammatory and antihyperalgesic	[205]
		Quercetin	Quercetin-methyl-β-cyclodextrin inclusion complex	• Improved solubility and purity	Potential anti-AD effects.	[206]
		Crocetin	Crorcetin-γ-cyclodextrin inclusion complex	• Enhanced bioavailability across blood-brain barrier	Treatment of AD	[207]
		Curcumin	Curcumin/hydroxypropyl-β-cyclodextrin inclusion complex	• Antioxidant and anti-inflammatory effect • Increased AUC values	Treatment of AD	[208]
3	Self-nano/ micro emulsifying drug delivery system (SNEDDS/ SMEDDS)	Fisetin	Fisetin-SNEDDS	• Improved oral bioavailability of fisetin • Low dose required for activity	Treatment of PD Anti-PD effects	[209] [210]
		Oxyresveratrol	Oxyresveratrol SMEDDS	• 4-Times reduction of dose	Treatment of AD	[211]
4	Nanospheres	Curcumin	Curcumin and selenium nanoparticles loaded nanospheres	• Enhanced therapeutic efficacy	Treatment of AD	[212]
		Amantadine	Amantadine-loaded methacrylate nanosphere	• Improved bioavailability	Treatment of ALS	[213]
5	Micelles	Linoleic acid	Lactoferrin-conjugated linoleic acid micelles	• Enhanced *in vivo* distribution in brain • Good hemocompatibility	Treatment of AD	[214]
		Curcumin	Curcumin loaded polymeric micelles	• Excellent intraocular biocompatibility	Diagnosis and management of AD	[215]
		Morin	Morin hydrate-loaded micellar nanocarriers	• High blood and brain drug concentration	Treatment of AD	[216]
		Hydroxytyrosol	Hydroxytyrosol-loaded Pluronic + dequalinium micellar nanocarriers	• Improved protection to cytotoxicity and oxidative stress	Anti-PD effects	[217]

**Table 2:** Details of various clinical trials completed/ongoing for natural products against neurodegenerative diseases.

**S. No.**	**Clinical Trial Number**	**Official Title**	**Intervention/** **Treatment Drug**	**Location(s)**	**Actual Enrollment**	**Phase Number**	**Status**	**Actual/ Anticipated Completion Date**	**References**
1	NCT00470418	Development of NIC5-15 in the treatment of Alzheimer's disease	NIC5-15	New York	15	2	Completed	March 2010	[218]
2	NCT04663854	Evaluation of therapeutic effects of trehalose in patients with Alzheimer's disease	Trehalose	Iran, Islamic republic of	20	1	Recruiting	August 2022	[219]
3	NCT01928420	A single site, randomized, double-blind, placebo-controlled trial of NIC5-15 in subjects with Alzheimer's disease	NIC5-15	New York	30	2	Completed	June 2014	[220]
4	NCT01190735	Caffeine for motor manifestations of Parkinson's Disease: An open-label, dose-response study	Caffeine alkaloid	Canada	28	2	Completed	February 2011	[221]
5	NCT02061878	A randomized controlled study to evaluate the effect of bexarotene- an RXR agonist – on beta-amyloid and apolipoprotein E metabolism in healthy subjects	Bexarotene	Florida	12	1	Completed	November 2014	[222]
6	NCT00963846	Huperzine for cognitive and functional impairment in Schizophrenia	Huperzine	Connecticut	56	2	Completed	December 2012	[223]
7	NCT01333865	A study of memantine hydrochloride (Namenda) for cognitive and behavioural impairment in adults with autism spectral disorders	Memantine	Massachusetts	25	4	Completed	July 2014	[224]
8	NCT04843813	Lutein and multiple sclerosis experimental studies (LuMES)	Dietary supplement: Lutein	Illinois	60	Not applicable	Recruiting	March 2023	[225]
9	NCT03476044	Effect of selenium of succinylcholine-induced postoperative myalgia after adult sinuscopic procedures	Selenium	Egypt	80	3	Completed	March 2020	[226]
10	NCT01022229	Phase 3 study of a compound natural health product in children with ADHD	Dietary supplement: Compound natural health product	Canada	16	3	Completed	November 2015	[227]
11	NCT04484454	ProdromeNeuro: An Open-Label Study of Omega 3 Oil Nutritional Supplementation for Aging-related cognitive decline	Dietary Supplement: ProdromeNeuro Omega 3 Oil Nutritional Supplement	California	20	Not applicable	Completed	March 2021	[228]
12	NCT05066503	Natural therapy for delirium in elderly hospitalized subjects	Dietary Supplement: 260279 active study product	Arkansas	45	Not applicable	Recruiting	March 2023	[229]
13	NCT03575676	Phase iia, double-blind, randomized, placebo-controlled study of the efficacy and safety of SOM3355 in J-Huntington disease patients with chorea movements	SOM3355	Spain	32	2	Completed	August 2019	[230]
14	NCT03761849	A randomized, multicenter, double-blind, placebo-controlled, phase iii clinical study to evaluate the efficacy and safety of intrathecally administered RO7234292 (rg6042) in patients with manifest Huntington's Disease	RO7234292	Albama	791	3	Recruiting	March 2022	[231]
15	NCT04000594	An open-label adaptive multiple-dose study to investigate the pharmacokinetics and pharmacodynamics of RO7234292 in csf and plasma, and safety and tolerability following intrathecal administration in patients with Huntington's Disease	RO7234292	Netherlands, London, and Manchester	12	1	Completed	January 2022	[232]
16	NCT02336633	Metabolic Intervention Using Resveratrol in Patients with Huntington's disease	Dietary supplement: Resveratrol	Paris	102	Not applicable	Completed	January 2020	[233]
17	NCT00980694	Bioavailability of ubiquinol in Huntington's disease	Dietary supplement: ubiquinol	New York	6	1	Completed	July 2012	[234]
18	NCT01458470	A trial of memantine as a symptomatic treatment for early Huntington Disease; a phase iib study	Memantine	Canada	19	2	Completed	November 2012	[235]
19	NCT04478734	Multicentric trial on the use of combined therapy of thiamine and biotine in patients with Huntington Disease	Moderate doses of thiamine y biotin	Spain	24	2	Not yet recruiting	June 2023	[236]
20	NCT01245530	Probable Alzheimer type dementia compare INM-176 1200~1600mg/day with donepezil 5~10mg/day of safety and efficacy to randomization, multicenter, double-blind, double-dummy, parallel phase iii clinical study	INM-176, Aricept	Korea, Republic of	280	3	Completed	March 2011	[237]
21	NCT02914769	Antidepressant effects of ayahuasca: a randomized placebo-controlled trial in treatment-resistant depression	Ayahuasca	Brazil	35	2	Completed	December 2016	[238]
22	NCT04785300	ALSENLITE: an open-label pilot study of senolytics for Alzheimer's Disease	Quercetin, and dasatinib	Minnesota	20	2	Enrolling by invitation	June 2024	[239]
23	NCT04685590	Phase ii clinical trial to evaluate the safety and feasibility of senolytic therapy in Alzheimer's Disease	Quercetin and dasatinib	North Carolina	48	2	Recruiting	January 2032	[240]
24	NCT02104752	Curcumin as a novel treatment to improve cognitive dysfunction in schizophrenia	Curcumin	California	39	2	Completed	October 2017	[241]
25	NCT01811381	Curcumin and yoga exercise effects in veterans at risk for Alzheimer's Disease	Curcumin	California	80	2	Not recruiting	December 2020	[242]

**Table 3:** List of patents granted for natural products against neurodegenerative diseases.

S. No.	Patent Title	Patent Claims	Year of Grant	Patent Number	References
1	Method of treating neurological conditions with oleandrin	Oleandrin contains composition to treat neurological conditions including stroke, HD, PD, AD, ALS, autism and diabetic neuropathy	January 2018	US 9877979 B2	[244]
2	Nutritional supplement for the prevention of cardiovascular disease, Alzheimer's disease, diabetes, and regulation and reduction of blood sugar and insulin resistance	A supplement in form of a drink or food comprising ten synergistic components (such as lycopene, naringenin, quercetin, hesperidin, diindolylmethane, glycine) capable of lowering the risk of AD	September 2011	US 8017147 B2	[245]
3	Pharmaceutical compositions containing phosphatidylserine and curcumin	A stable pharmaceutical composition or food supplement containing curcumin and phosphatidylserine has potential to treat brain aging-related disorders including AD	July 2016	US 9381204 B2	[246]
4	Luteolin and diosmin/diosmetin as novel STAT3 inhibitors for treating autism	Administering flavonoids to patients can treat schizophrenia and autism by inhibiting the activation of STAT3	July 2014	US 8778894 B2	[247]
5	Buccal and sublingual cannabinoid formulations and method of making the same	A troche formulation containing PEG, cannabis extract compound has the potential to treat AD, PD, HD, ADHD, depression, cancer, Crohn's disease, and ulcerative colitis	April 2021	EP 3160451 B1	[248]
6	Phenolic compositions derived from apple skin and uses thereof	Pharmaceutical composition having extract fraction of apple skin composing flavonol components (such as quercetin, quercetin-3-O-galactoside *etc*). The composition has the potential to treat autoimmune, neurodegenerative (AD, PD, and sclerosis), metabolic, and vascular disorders	December 2016	US 9511107 B2	[249]
7	Herbal composition for reducing ADD/ADHD and method thereof	An herbal formulation comprising herbal extracts to treat symptoms associated with ADHD	March 2013	US 8394429 B2	[250]
8	Nutraceutical composition that comprises an extract of Andean shilajit, for preventing and/or treating neurodegenerative diseases and/or the cognitive deterioration associated with cerebral aging	Nutraceutical formulation comprising a blend of shilajit, folic acid, vitamin B6, and B12. The composition has the potential to treat neurodegenerative diseases including AD, PD, and senile dementia	July 2014	US 8784804 B2	[251]
9	Cannabinoid-containing plant extracts as neuroprotective agents	Plant extract to be used for prevention of treatment of neurogenerative diseases including ALS, HD, PD, AD, and prion disease	March 2014	US 8673368 B2	[252]
10	Catechins for the treatment of fibrillogenesis in Alzheimer's disease, Parkinson's disease, systemic Aa amyloidosis, and other amyloid disorders	Green tea extract composing catechins for the treatment of amyloid diseases	January 2012	CA 2440293 C	[253]

## LIST OF ABBREVIATIONS

AChE: Acetylcholinesterase
AD: Alzheimer’s Disease
AGD: Argyrophilic Grain Disease
ALS: Amyotrophic Lateral Sclerosis
ASH: Amphid Single Neurons
BACE: β-site Amyloid Precursor Protein Cleaving Enzyme
BDNF: Brain Derived Neurotrophic Factor
BuChE: Butyrylcholinesterase
DAT: Dopamine Transporter
ERK: Extracellular-Signal-Regulated Kinase
FA: Friedreich Ataxia
GDNF: Glial Cell Line Derived Neurotrophic Factor
GSH: Glutathione
GSK: Glycogen Synthase Kinase
HD: Huntington’s Disease
hSOD: Human Superoxidase Dismutase
LPO: Lipid Peroxidation
MAO: Monoamine Oxidase
MDA: Malondialdehyde
mHTT: Mutant Huntingtin
MPTP: 1-Methyl-4-phenyl-1,2,3,6-tetrahydropyridine
NDDs: Neurodegenerative Disorders
3-NP: 3-Nitropropionic Acid
6-OHDA: 6-Hydroxydopamine
PD: Parkinson’s Disease
REB: Rat Endothelial Brain
SNCA: Synuclein Alpha
TFR: Transferrin Receptor Protein
UVR: Ultraviolet Radiation
